# Laboratory Fracture Resilience of Hybrid Abutments Used in Oral Rehabilitation: A Systematic Review

**DOI:** 10.3390/jfb13030120

**Published:** 2022-08-15

**Authors:** Luca Favasuli, Paulo S. Mascarenhas, Paulo Mauricio

**Affiliations:** Centro de Investigação Interdisciplinar Egas Moniz (CiiEM), Instituto Universitário Egas Moniz, Campus Universitário, Quinta da Granja, Monte de Caparica, 2829-511 Almada, Portugal

**Keywords:** hybrid abutment, dental implant, zirconia, screw, crown, cemented

## Abstract

When implants are required in prosthodontics treatment, one of the most important decisions is the choice of the final crown and the type of connection to the implant through the abutment. Hybrid abutments are becoming a primary choice. They are projected and produced with materials whose properties guarantee the required mechanical features (including resistance) and take advantage of the hybrid abutment crown retention between screw and cement. However, a review of the mechanical resistance of the different abutment types and associated materials is still lacking. This review aimed to study the in vitro mechanical efficiency of the hybrid abutments used in oral rehabilitation. Methods: A systematic review was conducted using the PubMed, B-on, and Google Scholar databases according to the Preferred Reporting Items for Systematic Reviews and Meta-Analyses (PRISMA) guidelines. Results: 75 articles were identified from all databases, and 33 were selected after abstract screening. Thus, 21 studies were included in the review after full-text reading. Among the materials used for crowns, lithium disilicate was, aesthetically, the primary choice for its aesthetic and moderate strength. On the other hand, zirconia showed the best fracture resistance. Regarding the different kinds of abutments, there is still some lack of knowledge about the best design. Conclusions: Within the limitations of this systematic review, we can conclude that hybrid pillars are an excellent choice for oral rehabilitation through implants, showing improved resistance when including materials such as zirconia and lithium disilicate.

## 1. Introduction

Reconstructions for problems of the dental arch supported by implants are considered the preferred option thanks to their favorable longevity, aesthetics, and comfort. One of the most important decisions in using dental implants in prosthodontics is the choice of the final crown and the type of connection to the implant through the abutment. The implant–crown abutment can be either cemented or screwed. Both connections have advantages and disadvantages. Before choosing the type of connection between the implant and the crown, the prosthetist should consider the outcomes and features of the crown, the type of abutment, and other issues described elsewhere [1]. Crowns held by cement have led to relevant biological involvements such as inflammation of soft tissues and pathological bone recession. Comparing the condition of the peri-implant soft tissues and the surrounding bone near the restoration by screw and cement, the results favour screwed restoration. Screwed crowns have many advantages, such as recoverability, better control of the soft tissue’s health, and limited height. Cemented restorations guarantee a better passive, aesthetic, and precision fit of the occlusal surface because it creates a more homogeneous load distribution during its function [1]. Hybrid abutment and ceramic crowns are new approaches to improve aesthetics and strength in dental implants, being a reliable option in the anterior region [2]. The mesostructures of the abutments are cemented in a middle extra-oral environment. They are cemented on a titanium base with a standard height and diameter and are available for different prosthetic connections [3]. The hybrid abutments are made of a titanium insert connected to a ceramic structure through resin cement [4]. In this modality, similar to natural tooth recovery, the abutment can be prefabricated or personalized and is attached to the crown by cement [5].

Today, through CAD/CAM technology, the hybrid abutment is projected and produced, with a focus on the mechanical features of the materials [6]. Currently, there is a wide range of CAD/CAM ceramic abutments with different geometries for connecting the abutment to the implant. The hybrid abutments provide many potential advantages: convenient mechanical features, simplicity, and efficiency [7]. These new systems have attracted remarkable interest due to their high resistance to fractures, pleasant aesthetics, accurate measures with the implant, and biocompatibility [8].

The hybrid abutment, where a bevelled edge is fixed to the palatal side and an intrasulcular component of the restoration is limited to the vestibular side, can be produced by two different designs:

- A hybrid abutment crown with the abutment and the crown fabricated as a unique piece that will be attached to the titanium base and screwed to the implant.
- A hybrid abutment with a separated crown with the abutment first fixed to the titanium base and then screwed to the implant, followed by cementation of the whole ceramic crown on it.

The hybrid abutment crown combines the retention advantages between the screw and cement. However, only a few studies have rated the differences between the hybrid crowns and the hybrid abutments with separated crowns [9,10].

Traditionally, hybrid abutments’ design and materials usually depend on the doctor’s and/or patient’s preferences and the patient’s clinical situation. However, mechanical efficiency is also important to consider. To our knowledge, there is still no review assessing the mechanical efficiency of the design and materials used in the hybrid abutments. Thus, we reviewed and synthesized, for the first time, the resilience results obtained from in vitro studies to contribute to the informed choice of abutment type in oral rehabilitation and to suggest improvements for future research.

## 2. Materials and Methods

The method used in this systematic revision met the PRISMA guidelines and was registered at PROSPERO (ID:341060). The PICO strategy was used to build the research questions:

Population: two-piece hybrid abutment (custom materials) with crown;
Intervention: laboratory mechanical stress tests (duration/strength/frequency);
Comparison: one-piece hybrid abutment (custom materials) with crown;
Outcome: hybrid abutment integrity or fracture rates.

This revision aims to evaluate the mechanical resilience of the hybrid abutments with crowns to in vitro mechanical stress tests.

### 2.1. Search Strategy

The research strategy included the electronic screening of English publications over the last 10 years on PubMed, Google Scholar, and B-on (included sources are listed at https://www.b-on.pt/colecoes/ (accessed on 1 August 2022)). The applied search algorithm was: “dental implant [MeshTerms] AND (hybrid abutment OR titanium base OR Ti-Base) AND crown AND zirconia AND (screw OR cemented)”.

The screening results were imported to Mendeley desktop citation manager software for deletion of replicates, and each of the remaining listings was selected if meeting the following criteria:

INCLUSION CRITERIA

- Articles from the last 10 years;
- English language;
- One- or two-piece hybrid abutments;
- Implant restoration;
- In vitro studies.

EXCLUSION CRITERIA

- Dental restoration;
- Cemented abutments;
- Screwed abutments.

### 2.2. Quality Assessment

We assessed the selected articles’ risk of bias according to the following domains: (D1) specimen randomization; (D2) single-operator protocol implementation; (D3) description of the sample size calculation; (D4) blinding of the testing machine operator; (D5) the presence of a control group; (D6) failure mode evaluation; and (D7) use of all materials according to the manufacturer’s instructions. These domains were adapted from the Sarkis–Onofre quality assessment tool [11].

### 2.3. Study Strategy

Two independent reviewers (L.F. and P.M.) first screened the titles and/or abstracts identified in the searches. The final selection of studies was independently performed by the same authors who reviewed the paper’s full text based on the inclusion criteria. Only studies that met the eligibility criteria were included for review. Discussion with a third reviewer (P.S.M.) resolved any disagreements or discrepancies.

## 3. Results

### 3.1. Search and Selection

**Figure 1:**
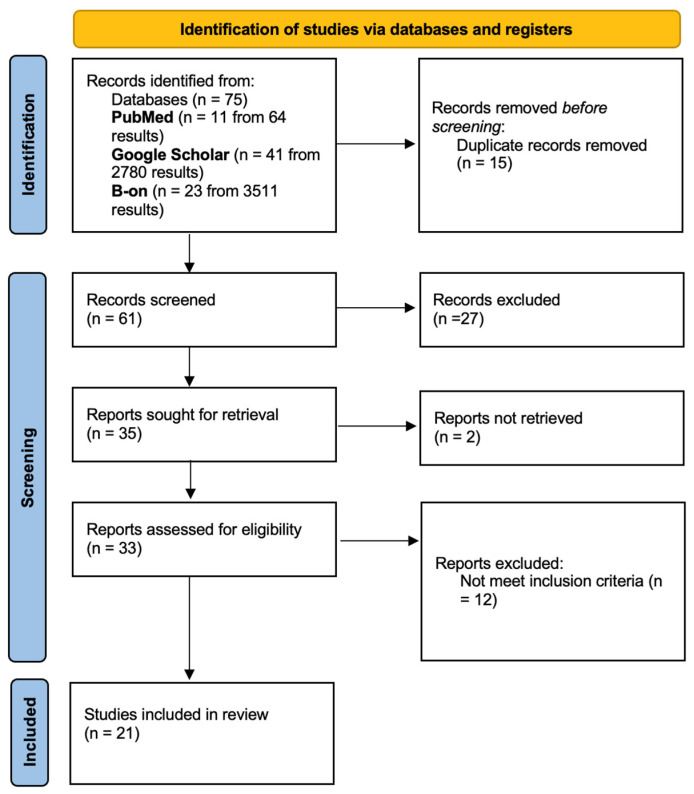
Flowchart for the search strategy according to the PRISMA statement.

A total of 75 articles were identified from all databases. No studies were identified from the grey literature. The Figure 1 flowchart summarizes the study selection process according to PRISMA. After removing duplicates, 61 manuscripts were identified for initial examination. Of these, 27 studies were excluded after titles and abstracts review. Two studies could not be retrieved for full-text analysis. Thus, 33 studies were chosen for full-text reading.

**Figure 2:**
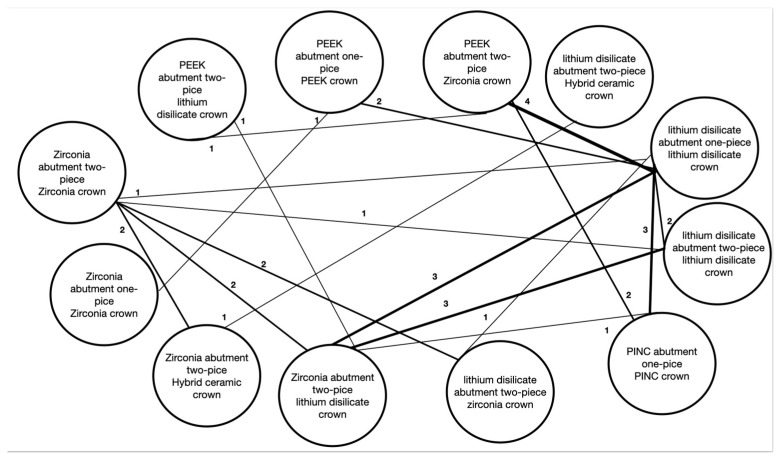
Hybrid abutment pairwise network. The line strength is proportional to the number displayed next to the line representing the amount of research comparing each pair of hybrid abutments in the review.

In Figure 2, we can observe that most studies compared one- or two-piece hybrid abutments of zirconia or lithium disilicate. Research on PICN and PEEK hybrid abutment is still scarce. The crowns used the most in these studies were built from lithium disilicate and zirconia. Only one study compared crowns made of hybrid ceramic against zirconia or lithium disilicate on different types of two-piece hybrid abutment materials. More studies about hybrid ceramics as hybrid abutment material are necessary to decide if it is a good alternative.

### 3.2. Description of Included Studies

Table 1 shows that two types of hybrid abutments (one-piece and two-piece) were used in this study. Only 5 of 21 studies effectively compared one- and two-piece hybrid abutments. The most used materials for these studies were zirconia and lithium disilicate. However, some authors decided to use alternative materials such as PINC and PEEK [10,12]. Regarding the materials used in the crowns, lithium disilicate was the most studied for its aesthetics and strength. On the other hand, zirconia has also been widely studied for its strength characteristics [13]. The Multilink^®^ Hybrid abutment was the most commonly used cement. However, other authors applied other resin types of cement. The abutment’s macrostructures of lithium disilicate were treated with hydrofluoric acid of different percentages. For the abutment’s macrostructures of zirconia, it was sandblasted with alumina particles in most cases or with alumina oxide particles. Due to the high heterogeneity of the pieces, used materials, and treatments, the quantitative results reported in articles were not extracted for meta-analysis. Other aspects related to the article’s results are dealt with within the Discussion section.

### 3.3. Risk of Bias

**Figure 3:**
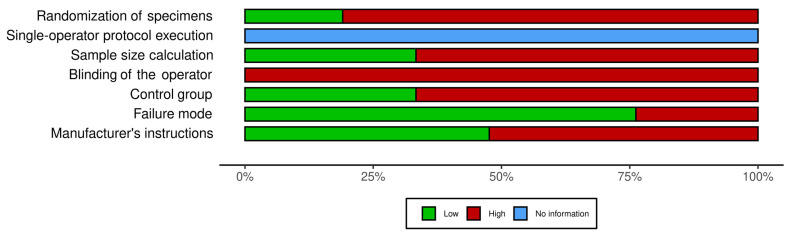
Risk of bias summary plot.

**Figure 4:**
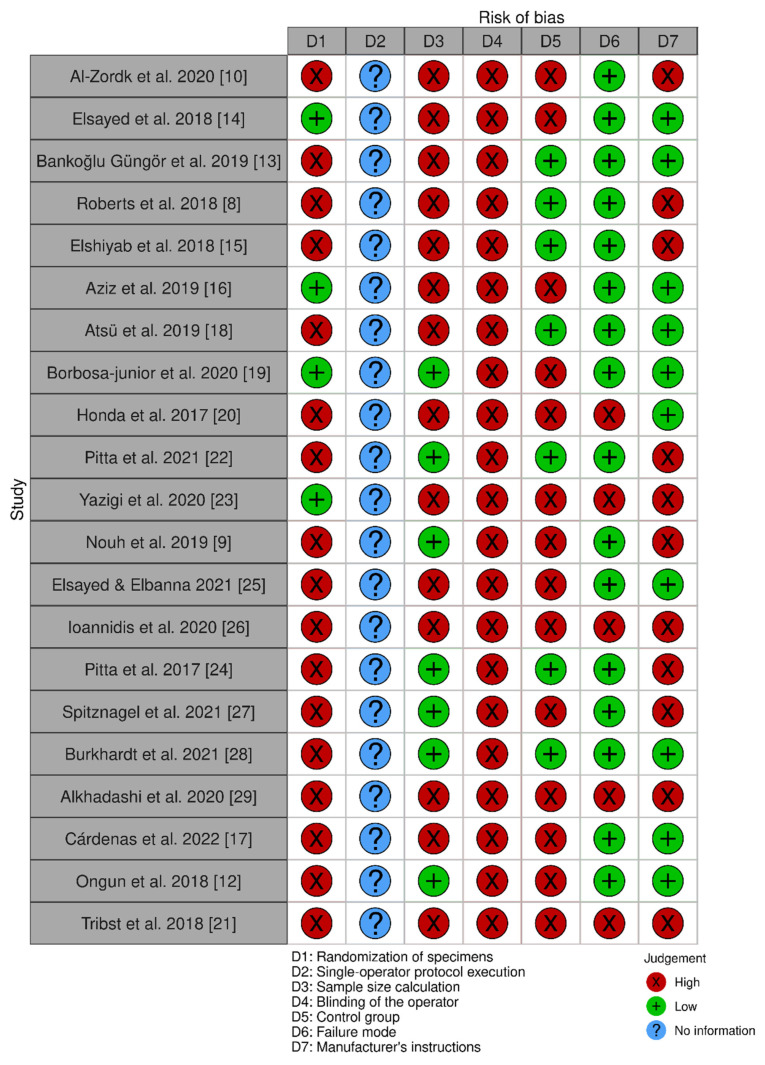
Risk of bias for included studies [8,9,10,12,13,14,15,16,17,18,19,20,21,22,23,24,25,26,27,28,29].

Figure 3 and Figure 4 show that the 22 articles used in this systematic review had a very high risk of bias, with only one parameter (failure mode) presenting a rating above 75% of low risk of bias. The main cause for risk of bias was that, in most studies, the reported mechanical efficiency was based on testing only one specimen of each hybrid abutment type.

## 4. Discussion

This systematic review enrolled hybrid abutments from in vitro studies. Many studies evaluated the resistance to fractures of materials with different kinds of hybrid abutments as a single piece or two-pieces with separated crowns. In these studies, the different kinds of materials for the crowns and the cementation protocols for the macrostructure of the titanium basis within the cementation of the crown above the macrostructure were also considered. Most authors have concluded that the zirconia hybrid abutment has the best fracture resistance. On the other hand, other authors, such as Elsayed et al. [14] and Roberts et al. [8], have reported that the disilicate lithium shows more fracture resistance and can be considered an alternative material to zirconia, especially in the anterior region with better aesthetic features. Nouh et al. [9], in their study of the comparison of different kinds of abutments, have concluded that the hybrid crown one-piece design has more defects than the separated crown hybrid abutment, probably because, in the latter, there is more strength split, even though titanium bases of 3 mm cannot simulate the mastication. Therefore, the base of titanium should be considered [9].

In studies with alternative materials such as PINC and PEEK, the authors have observed that the PINC material might not give enough fracture resistance due to the occlusal forces, even if it does not influence the titanium base or the screw [12,17]. PEEK shows more mechanical effort than zirconia regardless of the crown material. Therefore, PEEK shows a not significant difference in fracture resistance, even though it is considered an alternative material to zirconia [18,19]. In contradiction with previous studies, Al-Zordk et al. [10] have shown that the zirconia has a better fracture resistance than PEEK. Pitta et al. have also observed that the zirconia has significantly higher values of the bending moment than disilicate lithium and PINC, but the desilicated lithium presents better retention than zirconia and PEEK [24].

Regarding the differences between hybrid abutment types, Pitta et al. have observed in their study that the presence of a second interface between the hybrid abutment and the crown, and the absence of a canal for the screw on the abutments with separated crowns, can contribute to a better dissipation of the tension [22]. Tribst et al. [21] have confronted the combinations of different types of abutment materials with the different types of crown materials. This study shows that the elastic modulus of the ceramic crowns associated with an inferior elastic modulus of the abutment shows a better distribution of overall tension, suggesting a promising mechanic performance. Ioannidis et al. have observed a disadvantage that occurs in these hybrid abutments: the interface of the cementation of the crown on the abutment presents a significant gap, which can contribute to the development of periodontal disease. The dimension of the gap is influenced by the material of the hybrid abutment and the kind of interface [26].

In the in vitro studies, several authors observed different types of fractures or cementation failures during laboratory tests. These faults occurred mainly due to fracture of the titanium base, abutment fracture, failure of the cementation by deformation of the titanium base, and fracture of the crown. Nouh et al. [9] observed more failures in one-piece than two-piece hybrid abutments during the chewing simulation test. Elshiyab et al. [2] showed that the simulation of chewing over 5 years affected the resistance of different systems of fully ceramic crowns when submitted to implants, pointing out the mechanical and hydrothermal stress acceleration of the zirconia structures aging, and subsequently agreeing with the Bankoglu Gungor et al. study results [13]. Aziz et al. [16] observed in their experiments that the fractures were present only in the crowns and did not affect the hybrid abutment. In the lithium disilicate hybrid abutment, catastrophic fractures in the crowns and abutment while under masticatory forces were observed in the premolars zone. Therefore, it is an alternative option for hybrid abutment material, but is not the best choice for the premolars.

As stated before, the choice regarding the hybrid abutment type, applied material, and treatment is traditionally dependent on the clinical case, particularly on where the hybrid abutment will be placed. This review highlighted and discussed the mechanical efficiency of the whole set as an additional aspect of major importance and the aspects of current research that still need to be corrected or addressed in future studies.

## 5. Conclusions

Our review results show the following:

- Hybrid abutments are an adequate choice when mechanical resistance is an important requirement for implants, especially in the presence of materials such as zirconia and lithium disilicate. PICN and PEEK one- or two-piece hybrid abutments need more study;
- Limited evidence supports better mechanical performance in two-piece hybrid abutments due to a more balanced strength distribution;
- Overall, research in this field is still scarce. Furthermore, it is necessary to carry out more studies on the gap between the macrostructure and the crown and how much it can affect the periodontal tissues and might be a risk factor for the development of peri-implantitis;
- The mechanical testing research on hybrid abutments needs to adhere more thoroughly to quality assessment guidelines for in vitro studies to prevent the high risk of bias found in current studies for future research.

## Figures and Tables

**Table 1:** Detailed data regarding abutment type, material, treatment, and outcomes of the included studies.

Authors (Year)	Hybrid Abutments	Abutment Treatment	Crown Materials	Cement	Outcome
**Ongun et al. (2018)** [12]	Polymer-Infiltrated Ceramic-Network (PINC) (Vita Enamic, Vita Zahnfabrik, Bad Säckingen, Germany); (one-piece) Lithium disilicate (LDS) (IPS e.max CAD, Ivoclar Vivadent, Schaan, Liechtenstein) (one-piece)	Hydrofluoric acid 5% (HFA) gel (IPS Ceramic Etching Gel, Ivoclar Vivadent, Schaan, Liechtenstein)	Polymer-Infiltrated Ceramic-Network (PINC) Lithium disilicate	Multilink^®^ Hybrid Abutment (Ivoclar Vivadent, Schaan, Liechtenstein)	LDS was considered preferable to PICN for manufacturing customized hybrid abutment regarding bending, fracture, occlusal forces, and bonding resistance.
**Al-Zordk et al. (2020)** [10].	Zirconia (Z-CAD, Metoxit, Switzerland) (one-piece) PEEK (Bredent, Senden, Germany) (one-piece) Lithium disilicate (IPS e.max CAD, Ivoclar Vivadent, Liechtenstien) (one-piece)	Hydrofluoric acid 5% (LDS) (Ceramic Etchant, Dentobond, France) Airborne-particle abraded with 50 µm alumina oxide	Zirconia PEEK Lithium disilicate	Adhesive resin cement (DTK Adhesive, Bredent, LOT 476249)	Zirconia crown hybrid abutment showed more fracture resistance than hybrid pillar crown in PEEK and lithium disilicate.
**Elsayed et al. (2018)** [14].	Zirconia (two-piece) Lithium disilicate (IPS emax CAD; Ivoclar Vivadent, Schaan, Liechtenstein) (one and two-piece)	Hydrofluoric acid 4.5% (LDS) (IPS Ceramic Etching Gel, Ivoclar Vivadent) Airborne particle abraded with 50 µm alumina particles	Zirconia Lithium disilicate	Dual-curing luting composite resin (Multilink^®^ Automix Ivoclar Vivadent)	Hybrid one- and two-piece abutments made of lithium disilicate withstood high load forces with no difference in fracture resistance or failure mode. Using a titanium base in the hybrid abutment enhanced the strength of the zirconia abutments.
**Bankoğlu Güngör et al. (2019)** [13]	Zirconia (two-piece) Lithium disilicate (IPS e.max CAD, Ivoclar Vivadent, Schaan, Liechtenstein) (one and two-piece)	Hydrofluoric acid 5% (LDS) (IPS Ceramic Etching Gel, Ivoclar Vivadent) Airborne-particle abraded with 50 µm alumina oxide	Zirconia Lithium disilicate	Self-curing resin cement (Multilink^®^ Hybrid Abutment Ivoclar Vivadent)	Fracture resistance of lithium disilicate hybrid abutment was lower than zirconia hybrid abutment. Titanium bases increased resistance to fractures.
**Roberts et al. (2018)** [8]	Zirconia (Zyrcomat 6000 MS; Vita, Yorba Linda, CA, USA) (two-piece) Lithium disilicate (IPS e.max CAD, Ivoclar Vivadent, Schaan, Liechtenstein) (one and two-piece)	Hydrofluoric acid (IPS Ceramic Etching Gel) Airborne-particle abraded with 50 μm aluminum oxide	Zirconia Lithium disilicate	Autopolymerizing resin cement (Multilink^®^ Hybrid Abutment Ivoclar Vivadent) Dual-cure resin cement (Panavia™ F 2.0, Kuraray)	Lithium disilicate material was a viable alternative to zirconia as a hybrid abutment material. The lithium disilicate one-piece hybrid abutment had greater fracture resistance.
**Elshiyab et al. (2018)** [15]	Zirconia (Zenostar, Ivoclar Vivadent, Lichtenstein, Germany) (two-piece)	No information	Zirconia Lithium disilicate	Self-curing dental luting composite (Multilink^®^ Hybrid Abutment Ivoclar Vivadent)	Monolithic crowns of zirconia had significantly higher fracture resistance than those of lithium disilicate.
**Aziz et al. (2019)** [16]	Zirconia Yttria stabilized polycrystalline zirconia (Y-TZP) (Katana zirconia) (two-piece) Lithium disilicate (IPS e.max CAD, Ivoclar Vivadent, Schaan, Liechtenstein) (two-piece)	Hydrofluoric acid 9,5% gel (Bisco) Airborne-particle abraded with 50 µm aluminum oxide	Zirconia Lithium disilicate	Totalcem self-etch/Self-adhesive Resin cement (ITENA, Villepinte, France)	Zirconia hybrid abutment had a significantly higher fracture resistance.
**Cárdenas et al. (2022)** [17]	Zirconia (Straumann CARES; Institut Straumann AG, Basel, Switzerland) (two-piece) Lithium disilicate (IPS e.max CAD, Ivoclar Vivadent, Schaan, Liechtenstein) (one-piece) PICN (Enamic; VITA Zahnfabrik) (one-piece)	Hydrofluoric acid 5% (IPS ceramic etching gel; Ivoclar Vivadent AG) Airborne-particle abraded with 50 µm aluminum oxide	Zirconia Lithium disilicate PINC	Resin-based cement (Multilink^®^ Hybrid Abutment Ivoclar Vivadent)	Although failures were catastrophic, the two-piece hybrid abutment endured higher fatigue resistance values. The hybrid abutment in PICN had the lowest fracture resistance. However, the failure did not affect base nor screw. Hybrid abutment in PICN was not recommended for the anterior teeth due to limited survival. The lithium disilicate and two-piece zirconia abutment had a much greater fatigue resilience than a lithium disilicate piece and were recommended as an aesthetical alternative for restoring a single implant in the anterior region.
**Atsü et al. (2019)** [18]	Zirconia (two-piece) RPEEK (BioHPP, SKY implant, Bredent, Germany) (two-piece)	Airborne-particle abraded with 110 µm aluminum oxide	Lithium disilicate (IPS e.max CAD, Ivoclar Vivadent, Schaan, Liechtenstein)	Adhesive resin cement (Panavia™ V5 Kuraray Noritake Dental Inc., Tokyo, Japan)	Zirconia and RPEEK had similar fracture resistance. RPEEK had the potential to withstand maximum occlusal forces in the anterior area.
**Borbosa-junior et al. (2020)** [19]	Zirconia (Prettau Translucent, Zirkonzahn) (two-piece) PEEK (Dental PEEK disk Tecno Med Mineral, Zirkonzahn) (two-piece)	Airborne-particle abraded with 50 µm aluminum oxide	Zirconia Lithium disilicate (IPS e.max CAD, Ivoclar Vivadent, Schaan, Liechtenstein)	Self-adhesive resin cement (Relyx™ U200, 3M ESPE)	Hybrid abutment made of PEEK had similar mechanical fatigue compared to Zirconia, regardless of the material in the crown. Crowns of transparent zirconia presented superior mechanical fatigue compared to lithium disilicate crowns used with zirconia abutments. Lithium disilicate crowns had mechanical fatigue similar to translucent zirconia when used with custom PEEK abutments.
**Honda et al. (2017)** [20]	Porcelain layer zirconia-based restorations (PLZ) (Katana Zirconia; Kuraray Noritake Dental Inc.) (one-piece) Indirect zirconia-based composite layer restorations (ILZ) (Katana Zirconia; Kuraray Noritake Dental Inc.) (one-piece) Metal-ceramic (MC) monolithic zirconia (MONO) (Katana Zirconia; Kuraray Noritake Dental Inc.) (one-piece)	Airborne-particle abraded with 50 µm aluminum oxide	Zirconia	Dual-polymerized resin material (Panavia™ F2.0, Kuraray Noritake Dental Inc., Tokyo, Japan)	The fracture resistance of ILZ restorations was comparable to that of PLZ and MC restorations. Fracture loads were significantly higher for monolithic zirconia restorations than for layered restorations. All restorations were able to support masticatory molar physiological forces.
**Tribst et al. (2018)** [21]	Zirconia (InCoris ZI meso, Sirona, Dentsply Sirona, São Paulo, Brazil) (two-piece) Lithium disilicate (IPS e.max CAD Abutment Solutions, Ivoclar Vivadent, Schaan, Liechtenstein) (two-piece) Hybrid ceramic (VITA Enamic Implant Solutions, VITA Zahnfabrik, Bad Säckingen, Germany) (two-piece)	No information	Zirconia Lithium disilicate Hybrid ceramic	No information	Lower stress concentration was observed using a material with a higher elastic modulus. Zirconia crowns promoted a lower chance of catastrophic failure in the cement line between the crown and the hybrid abutment. The combination of a ceramic crown with high modulus elastic in contact with the load application, and then a material with smaller elastic modulus under the crown, mimics the behaviour of enamel and dentin. The hybrid ceramic abutment presented a better strain standard and improved the strain distribution in the first cement line, thus making it the most suitable material. The ceramic crowns’ upper elastic modulus associated with the hybrid abutment’s lower elastic modulus showed a better distribution of stresses in the assembly improving mechanical behaviour.
**Pitta et al. (2021)** [22]	Zirconia (Lava Plus, 3M, St. Paul, MN, USA) (one-piece) Lithium disilicate (IPS e.max CAD Abutment Solutions, Ivoclar Vivadent, Schaan, Liechtenstein) (one-piece) PINC (Vita, Enamic, Vita Zahnfabrik, Bad Säckingen, Germany) (one-piece)	Hydrofluoric acid 5% (IPS ceramic etching gel, Ivoclar Vivadent AG, Schaan, Liechtenstein) Airborne-particle abraded with 50 µm aluminum oxide	Zirconia Lithium disilicate PINC	MDP monomer-containing resin cement (Panavia™ 21 Kuraray, Tokyo, Japan)	Hybrid abutment crowns in zirconia and PICN revealed lower survival and higher complication rates than restorations in the other studies. The presence of a second interface between a hybrid abutment and crown and the lack of access to screw through the crown can contribute to a better dissipation of tension, unlike a single interface between the crown abutment and titanium base with access to screw as in the hybrid abutment restoration crown.
**Yazigi et al. (2020)** [23]	Zirconia (IPS e.max ZirCAD, Ivoclar Vivadent, Schaan, Liechtenstein), (one-piece) Lithium disilicate (IPS e.max CAD Abutment Solutions, Ivoclar Vivadent, Schaan, Liechtenstein (one-piece) PICN (Vita Enamic, Vita Zahnfabrik, Bad Säckingen, Germany) (one-piece) RPEEK ioHPP elegance prefab blocks, Bredent) (one-piece) Nano-hybrid composite resin (Grandio blocs, Voco, Cuxhaven, Germany) (one-piece)	Hydrofluoric acid 5% (IPS Ceramic Etching Gel, Ivoclar Vivadent) Airborne-particle abrasion with 50 µm (zirconia) Airborne-particle abrasion with 50 µm aluminum oxide (hybrid composite resin)	Zirconia Lithium disilicate PICN RPEEK Nano-hybrid composite resin	Dual curing resin cement (DTK-Kleber, Bredent)	Zirconia presented the greatest resistance to fracture and the nano-hybrid resin presented the least resistance to fracture and did not withstand the physiological occlusal loads. RPEEK presented greater fracture resistance than lithium disilicate, but lower than zirconia, so it may be recommended as an alternative to restore single implants in the posterior area.
**Nouh et al. (2019)** [9]	Zirconia (Zenostar Zr Translucent; Wieland Dental, Pforzheim, Germany) (one and two-piece) Lithium disilicate (IPS e.max CAD; Ivoclar Vivadent, Schaan, Liechtenstein) (one and two-piece	5% hydrofluoric acid (IPS Ceramic Etching Gel, Ivoclar Vivadent) Airborne-particle abrasion with 50 µm (zirconia)	Zirconia Lithium disilicate	Self-curing resin cement (Multilink^®^ Hybrid Abutment Ivoclar Vivadent)	One-piece hybrid abutment in zirconia had a few more failures than hybrid abutment with separated crowns in zirconia. This may be due to better dissipation of forces following the presence of multiple interfaces. Hybrid abutment with a separated corona in lithium disilicate presented more faults than the one-piece hybrid abutment in lithium disilicate, which can be attributed to the greater resistance of the material when used as a monolithic block. Zirconia and lithium disilicate (hybrid abutment crowns and hybrid abutment with separated crown) with short titanium bases (3 mm) failed to simulate chewing. Therefore, despite its high resistance to fracture, its use in the posterior region should be avoided.
**Pitta et al. (2017)** [24]	Zirconia (Lava Plus, 3M, St. Paul, MN, USA) (two-piece)	3M 30 µm (3M ESPE)	Lithium disilicate (IPS e.max CAD; Ivoclar Vivadent, Schaan, Liechtenstein) PICN (Vita, Enamic, Vita Zahnfabrik, Bad Säckingen, Germany) Zirconia	Resin cement containing MDP (Panavia™ 21 Kuraray, Tokyo, Japan)	Zirconia presents significantly higher bending moment values than lithium-disilicate and PICN groups. All ceramics (lithium disilicate, zirconia and PICN) had very good stability when used in their monolithic form.
**Elsayed & Elbanna (2021)** [25]	Zirconia (Zolid HT, Ammann Girrbach, Austria) (one-piece) Lithium disilicate (IPS e.max CAD; Ivoclar Vivadent, Schaan, Liechtenstein) (one-piece) PEEK (BreCAM BioHPP, Bredent GmbH, Germany) (one-piece)	Hydrofluoric acid 5% (IPS Ceramic Etching Gel, Ivoclar Vivadent, Lichtenstein) Airborne-particle abrasion with 50 µm (zirconia) Airborne-particle abrasion with 110 µm (PEEK)	Lithium disilicate PEEK Zirconia	Dual curing resin cement (DTK-Kleber, Bredent)	Lithium disilicate presented greater retentivity compared to zirconia and PEEK. There was no significant difference between zirconia and PEEK.
**Ioannidis et al. (2020)** [26]	Lithium disilicate (IPS e.max CAD; Ivoclar Vivadent, Schaan, Liechtenstein) (one-piece) PICN (Vita Enamic, Vita Zahnfabrik, Bad Säckingen, Germany) (one-piece)	Hydrofluoric acid 5% (IPS Ceramic Etching Gel, Ivoclar Vivadent, Lichtenstein)	Lithium disilicate PICN	Self-curing resin cement (Multilink^®^ Hybrid Abutment Ivoclar Vivadent)	The interface for cementation of the crown on the abutment presented a large percentage of space, contributing to periodontal disease development. The restorative material’s composition and the interface’s nature influence the dimension of space.
**Spitznagel et al. (2021)** [27]	Lithium disilicate (IPS e.max CAD; Ivoclar Vivadent, Schaan, Liechtenstein) (one and two-piece)	Hydrofluoric acid 4.9% (IPS Ceramic Etching Gel, Ivoclar Vivadent, Lichtenstein)	Lithium disilicate	Self-curing resin cement (Multilink^®^ Hybrid Abutment Ivoclar Vivadent)	Hybrid abutment with a separated cemented crown dissipated better the physiological forces of chewing. Hybrid abutment (one-piece) had significantly better results.
**Burkhardt et al. (2021)** [28]	Lithium disilicate (IPS e.max CAD; Ivoclar Vivadent, Schaan, Liechtenstein) (one-piece)	Hydrofluoric acid 5% (IPS Ceramic Etching Gel, Ivoclar Vivadent, Lichtenstein)	Lithium disilicate	Auto-polymerization composite (Multilink^®^ Hybrid Abutment Ivoclar Vivadent)	Retention forces of lithium disilicate crowns on the hybrid abutment were influenced by salivary contamination and titanium surface cleaning. However, salivary contamination followed by a cleaning procedure decreased retention forces compared to non-contamination. There was only a significant reduction for contaminated surfaces cleaned with alcohol.
**Alkhadashi et al. (2020)** [29]	Lithium disilicate (IPS e.max press; Ivoclar Vivadent, Schaan, Liechtenstein) (one-piece)	Hydrofluoric acid 9.5% Gel (Bisco) Airborne-particle abraded with 50 µm alumina oxide	Lithium disilicate	Multilink^®^ Hybrid Abutment Ivoclar Vivadent Panavia™ SA Cemnent Plus, Kuraray	Abrasion of alumina particles yielded greater binding resistance than hydrofluoric acid conditioning 9.5% regardless of the cementation procedure. The two cements used were significantly different when subjected to particle abrasion or conditioning with hydrofluoric acid. The surface treatment of titanium alloys significantly influenced the shear binding force at the interface between titanium alloy and lithium disilicate.
